# A regulatory cascade of three transcription factors in a single specific neuron, DVC, in *Caenorhabditis elegans*

**DOI:** 10.1016/j.gene.2011.11.042

**Published:** 2012-02-15

**Authors:** Huiyun Feng, John S. Reece-Hoyes, Albertha J.M. Walhout, Ian A. Hope

**Affiliations:** a Institute of Integrative and Comparative Biology, Faculty of Biological Sciences, The University of Leeds, Leeds, LS2 9JT, UK; b Program in Gene Function and Expression and Program in Molecular Medicine, University of Massachusetts Medical School, 364 Plantation Street, Worcester, MA 01605, USA

**Keywords:** AD, activation domain, ANOVA, analysis of variance, Bp, base pairs, ChR2, channelrhodopsin-2, DAPI, 4′,6-diamidino-2-phenylindole, DB, DNA binding domain, DIC, differential interference contrast, DNA, deoxyribonucleic acid, EST, expressed sequence tag, FITC, fluorescein isothiocyanate, GFP, green fluorescent protein, Hr, hour, IPTG, isopropyl β-d-1-thiogalactopyranoside, modENCODE, model organism encyclopaedia of DNA elements, NGM, nematode growth media, NTP, nucleotide triphosphate, ORF, open reading frame, PCR, polymerase chain reaction, RNA, ribonucleic acid, RNAi, RNA interference, TF, transcription factor, UTR, untranslated region, Y1H, yeast one hybrid, Y2H, yeast two hybrid, YFP, yellow fluorescent protein, *Caenorhabditis elegans*, Homeodomain transcription factors, Neural gene expression, *ceh-63*, *ceh-14*, *mbr-1*

## Abstract

Homeobox proteins are critical regulators of developmental gene transcription and cell specification. Many insights into transcriptional regulation have been gained from studies in the nematode *Caenorhabditis elegans*. We investigated the expression and regulation of the *C. elegans* homeobox gene *ceh-63*, which encodes a single-homeodomain transcription factor of 152 amino acids. *ceh-63* is expressed in the interneuron DVC in both sexes, from late embryogenesis through adulthood, and two pairs of uterine cells in reproductive hermaphrodites only. A reporter gene fusion, encoding GFP fused to the full-length CEH-63, also drove weak inconsistent expression in additional unidentified cells in the head and tail. A potential *ceh-63* null mutant had no obvious abnormalities, except for a possible increase in subtle defects of the DVC axon projection. No behavioural responses were observed upon either laser ablation of DVC or activation of DVC through light stimulation of channelrhodopsin-2 specifically expressed in this neuron. The function of DVC therefore remains enigmatic. A transcriptional regulatory cascade operating in DVC was defined from the LIM-homeodomain protein CEH-14 through CEH-63 to the helix–turn–helix transcription factor MBR-1. Both CEH-14 and CEH-63 individually bound the *mbr-1* promoter in a yeast one-hybrid assay. A model is proposed suggesting that CEH-14 activates *ceh-63* and then along with CEH-63 co-ordinately activates *mbr-1*.

## Highlights

► The *C. elegans* homeobox gene *ceh-63* is expressed in a single interneuron, DVC. ► Although a highly conserved gene, a *ceh-63* null mutant had no major phenotype. ► A transcription factor regulatory cascade operating in DVC was defined.

## 1 Introduction

In most animals, the nervous system is by far the most complex tissue raising fundamental questions about how each nerve cell identity is specified, how the axon of each cell follows the appropriate trajectory and how each synaptic connection is correctly established. The neuroanatomy of the nematode *Caenorhabditis elegans*, however, is described completely, from electron microscope serial section reconstructions, down to the level of individual synaptic connections (Bargmann, 2006; Chalfie et al., 1985; Hall and Russell, 1991; White et al., 1986). The adult hermaphrodite has precisely 302 neurons in 118 distinct classes. These questions can therefore, in *C. elegans*, be addressed for specifically identified individual nerve cells.

Regulatory *trans*-acting factors, e. g. transcription factors and microRNAs, acting combinatorially, are known to be critical determinants of neuronal cell fate specification (Hobert, 2004; Johnston et al., 2005). Transcription factors often act hierarchically, in a network, to confer a progressive restriction in the developmental potential of a neuronal subtype until terminal differentiation is established (Altun-Gultekin et al., 2001). The homeodomain family of transcription factors controls neuronal identities in both spatial and temporal domains, and homeodomain-transcription factor networks directing the specification of various neuronal subtypes in *C. elegans* have been described (Altun-Gultekin et al., 2001; Hobert, 2005; Hobert et al., 1998; Sarafi-Reinach et al., 2001; Tsalik et al., 2003). There are approximately 100 homeodomain transcription factor genes in the entire *C. elegans* genome (Reece-Hoyes et al., 2005).

Anatomical gene expression patterns, as revealed by reporter gene fusions, allow visualization, *in vivo*, of dynamic differential gene expression through neuronal specification. We have previously investigated the expression patterns of promoter-GFP fusion genes for 366 of the approximately 940 *C. elegans* transcription factor genes (Reece-Hoyes et al., 2007). Transgenic lines were generated by microparticle bombardment transformation (Praitis et al., 2001) using reporter gene fusions created by high-throughput Gateway recombinational cloning (Hartley et al., 2000) based on the Promoterome resource (Dupuy et al., 2004). A large proportion of these transcription factor gene promoters drove neuronal expression, reflecting the complexity of the *C. elegans* nervous system and the major role of transcription factors in the development of this tissue. Most expression patterns involving the nervous system were complex with GFP seen in many nerve cells and other tissues. However, the expression pattern driven by the promoter of one homeobox gene, then known as *C02F12.5* and now known as *C02F12.10* or *ceh-63* was strikingly simple consisting of a single nerve cell in the tail plus some weaker expression in the wall of the uterus (Reece-Hoyes et al., 2007).

**Fig. 1:**
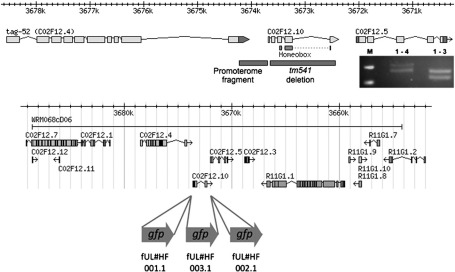
*ceh-63 / C02F12.10* gene structure with flanking genes. The intron / exon structures of the gene models as presented in WormBase are indicated. The untranslated regions, as previously defined by experiment, are in dark grey. The part of the *ceh-63* coding region encoding the homeodomain and deleted in the *tm541* allele are also indicated as is the region cloned in the Promoterome for this gene. The scales in kilobase pairs refer to position along the X chromosome. Inset: *ceh-63* cDNA fragments derived by PCR amplification of templates, in the *C. elegans* cDNA library, either containing (top band) or lacking (bottom band) intron 3. Products generated using primers For1 and Rev4 (1–4) or For1 and Rev3 (1–3) were resolved by agarose gel electrophoresis. (Primer sequences are provided in Supplementary Table 1.) DNA size markers (M) are 300 bp (top) and 150 bp (bottom). In the lower part of the figure, the genomic region contained in the fosmid WRM068cD06 is presented. The *gfp* reporter was inserted precisely at the start (fUL#HF001.1) or end (fUL#HF002.1) or to replace (fUL#003.1) the *ceh-63* protein-coding region by recombineering.

With the characterization of *C02F12.5* ESTs, the gene initially annotated as *C02F12.5* in WormBase was subsequently annotated as two separate genes, *C02F12.10* and *C02F12.5* (Fig. 1). It is *C02F12.10* that contains the homeobox and for which the expression driven by the promoter had been assayed with the promoter::*gfp* fusion (Reece-Hoyes et al., 2007). *C02F12.10* has now been given the genetic gene name *ceh-63*. The absence of experimental data for the *ceh-63* gene model meant that *ceh-63* transcripts had to be characterized first before defining the *ceh-63* expression more precisely. We determined the identity of the nerve cell in which this gene is primarily expressed, investigated the function both of this nerve cell and of *ceh-63*, and defined a cascade of transcription factors working through CEH-63.

## 2 Materials and methods

### 2.1 C. elegans strains

All strains were maintained at 20°C on 5 cm NGM agar plates seeded with *Escherichia coli* OP50 as food source (Sulston and Hodgkin, 1988). *C. elegans* N2 (Bristol) was used as wild type. Transgenic *C. elegans* strains utilised were: TB513{*dpy-20*(*e2017*)*IV;chIs513*[*pHK107*(*ceh-14*(*1*^*st*^*exon*)*-gfp-w/o-NLS*)*,dpy-20*(*+*)]*V*} (Cassata et al., 2000), PY2016{*OyIs32*[*lin-11::gfp*]} (Sarafi-Reinach et al., 2001), UL2650/2651/2652{*unc-119*(*ed3*)*III; pUL#JRH10H1*[*CO2F12.10*^*prom*^*::gfp, unc-119*(*+*)] (Reece-Hoyes et al., 2007) and an unnamed strain with the integrated transgene *Is*[*mbr-1*^*prom*^*::gfp*] (Kage et al., 2005). Mutant *C. elegans* strains included UTK2 {*mbr-1*(*qa5901*)} (Kage et al., 2005), TB528 [*ceh-14*(*ch3*) *X*] (Cassata et al., 2000) and an unnamed strain with *ceh-63*(*tm541*) (http://www.shigen.nig.ac.jp). The *qa5901* deletion removes the first half of the protein-coding region plus the promoter region and is likely to be a null allele of *mbr-1*. The *ch3* allele causes a translational frameshift upstream of the homeodomain and is a null allele of *ceh-14*, as confirmed with genetic data (Cassata et al., 2000).

To examine *ceh-63::gfp* expression in the absence of *ceh-63* function, the transgenic extrachromosomal array *leEx2650*[*ceh-63*^*prom*^*::gfp, unc-119*(*+*)] was crossed from strain UL2650 into the *ceh-63(tm541)* mutant background, both of the original strain, giving strain UL3105, and of the backcrossed strain, UL3122, giving strain UL3551. In addition, the extrachromosomal array *leEx3025*, carrying the recombineered fosmid with the *ceh-63* protein coding region replaced with *gfp*, in the strain UL3025 was also crossed into the *ceh-63(tm541)* mutant background of UL3122, giving strain UL3161. To examine *ceh-63::gfp* expression in the absence of *ceh-14* function, the *leEx3025* transgene was also crossed into the *ceh-14(ch3)* mutant background.

### 2.2 PCR amplification of ceh-63 cDNA

Sequences of PCR primers, for amplification of *ceh-63* cDNA fragments from a mixed-stage *C. elegans* cDNA library (Walhout et al., 2000), were based on the predicted gene model and flanking regions of the vector (pPC86) or directed at the poly-A tail (Supplementary Table 1). PCR was performed in 1 × BioTaq buffer, 2.5 mM MgCl_2_, 0.5 mM dNTPs, 0.5 μM of each primer, 0.25 units / μl of BioRed Taq enzyme, and 0.5 ng / μl purified cDNA library DNA. The PCR program consisted of 94°C 2 min, 30 cycles of 94°C 30 sec, 55°C 45 sec, and 72°C 2 min, and a final incubation at 72°C for 10 min. PCR products were either separated by agarose gel electrophoresis with distinct bands purified for sequencing or cloned into the pGEM-T-Easy cloning vector (Promega, USA) with screening by PCR before sequencing.

### 2.3 Recombineering of reporter gene fusions

Recombineering of *C. elegans* fosmid clones was carried out according to Bamps and Hope (2008). Primer sequences are provided in Supplementary Table 1.

### 2.4 Expression pattern analysis

A Leica DMR HC microscope fitted with GFP (Chroma 41017), YFP (Chroma 51017), and DAPI/FITC/TexasRed (Chroma 61002) filter sets was used to inspect the reporter expression patterns and identify neuronal identities by DIC. A Hamamatsu ORCA-ER B/W CCD camera was used to capture images with an Improvision Openlab imaging processing system. A Zeiss LSM 510 confocal system was used to capture z-stacks at 1 μm intervals. Improvision Volocity was used to visualize, organize and export images from Openlab and the confocal microscope. Reporter gene expression was observed in many hundreds of individual transgenic animals of mixed ages representing the entire life-cycle apart from the more specific observations for which, as stated in the Results section, only adult hermaphrodite or males were examined.

### 2.5 Behavioural assays

The defecation motor cycle of well-fed, healthy adult worms was measured manually under 100 × magnification at room temperature. Only the posterior body wall muscle contraction (pBoc) and expulsion muscle contraction were followed. The time of each expulsion was recorded over two 10 min windows for each worm, 5 worms for each strain.

Brood size was measured by picking L4 animals individually to fresh culture plates, with subsequent serial transfer at 24-hr intervals. The fertilized eggs laid in each 24-hr period were counted until no more eggs were laid. The total number of eggs laid by each hermaphrodite was taken as the brood size.

To compare growth rates, synchronized L1s, hatched overnight on culture plates without bacterial food, were transferred onto a fresh 5 cm NGM plate with an OP50 bacterial lawn grown from 500 μl of an overnight culture and maintained at 20°C. Their development was monitored everyday recording on which day all the bacteria was consumed. At 40 h after L1s had resumed development upon supply of bacterial food, the numbers of animals in late L3 / early L4, late L4, and young adult stage were counted.

To measure life span, about 200 L1s hatched out during a 2-hr period were left to grow to the L4 stage on culture plates at 20°C. These L4s were then distributed to fresh seeded plates, 10 per plate. Individuals were transferred to new plates every day during their reproductive period and then examined every day until their death, as determined by lack of movement even in response to physical stimulation. Each day, dead individuals were removed from the plates and the deaths were recorded.

Statistics analyses of the behavioural assays were performed using one-way ANOVA in OriginPro7.5 (Origin Lab Corporation).

### 2.6 Laser ablation of DVC

The UL2650 transgenic strain was used for laser ablation as its GFP expression facilitated identification of DVC and served as a marker to evaluate the effectiveness of the ablation. Laser ablation was performed with a pulsed microbeam laser, focused through the 100 × objective, on a Zeiss Axioscope equipped with Nomarski and epifluorescent optics. Newly-hatched L1 animals were mounted in 10 μM sodium azide on freshly prepared 3% agar pads under a coverslip. The GFP in DVC was bleached after just a few laser pulses, but 20–40 pulses were delivered to the DVC nucleus until it was seen shrunken and collapsed. After ablation, the worm was allowed to recover in M9 buffer on the agar pad for 10 min before being transferred with a glass pipette to a culture plate with OP50 bacteria as food source. Operated worms were grown for 3 days and examined for abnormalities in locomotion before being mounted to verify the absence of DVC. Control animals were subjected to the same procedure except that no laser pulses were applied.

### 2.7 Optogenetic analysis

Promoter::*ChR2::yfp* fusions were generated by Multisite Gateway cloning (Hartley et al., 2000). Promoter fragments were amplified by PCR using attB4 attached forward and attB1r attached reverse primers. Fosmids WRM068cD06 and WRM0620bD04, and plasmid *myo-3*^*prom*^*::ChR2::yfp* (non-Gateway version) (Nagel et al., 2005) were used as the templates for amplification of the *ceh-63*, *ceh-19b* and *myo-3* promoters, respectively. The channel rhodopsin *ChR2* coding sequence was amplified from the non-Gateway *myo-3*^*prom*^*::ChR2::yfp* plasmid (Nagel et al., 2005) with attB1 attached forward and attB2 attached reverse primers. The coding sequence of *yfp*-unc54 3′UTR was amplified by PCR using attB2r attached forward and attB3 attached reverse primers on the same plasmid. BP reactions between PCR fragments and appropriate pDONR vectors generated pENTRY-L4-*ceh-63*^*prom*^-R1, pENTRY-L4-*myo-3*^*prom*^-R1, pENTRY-L4-*ceh-19b*^*prom*^-R1, pENTRY-L1-*ChR2*-L2, and pENTRY-R2-*yfp*-*unc-54* 3′UTR-L3 entry clones. Entry clones for *ChR2* and *yfp* were examined by sequencing to check that no errors were incorporated during the PCR amplifications. LR reactions between entry clones and pDEST-R4-R3 generated the desired plasmids.

Transgenic strains were generated by microinjection for each of the *promoter::ChR2::yfp* fusions and expression patterns were examined. Promoters from *ceh-63*, *myo-3*, and *ceh-19* drove expression of ChR2-YFP specifically in DVC, body wall muscles or the pharyngeal MC neurons, respectively, as intended. A few adult hermaphrodites were transferred to NGM culture plates, supplemented with 5 mM all-trans-retinal and maintained in the dark. After 3–4 days, adult progeny were picked into 5 μl H_2_O on agarose pads. Blue light (475 nm) provided by a monochromator filtered through the Chroma Endow GFP filter on a Leica DMR HC microscope was used to activate the ChR2 under a 40 × objective lens and the behaviour of the illuminated animal was followed. As expected, strong instant simultaneous body wall muscle contraction was observed upon blue light illumination of worms transgenic for the *myo-3*^*prom*^*::ChR2::yfp* fusion, as previously reported (Nagel et al., 2005). Blue light illumination elicited pharyngeal pumping specifically, in the absence of bacteria, in animals transgenic for *ceh-19*^*prom*^*::ChR2::yfp*.

### 2.8 RNAi by feeding

RNAi by feeding was carried out as described by Kamath and Ahringer (2003). Standard NGM plates were supplemented with ampicillin (50 μg/ml), tetracycline (10 μg/ml), and IPTG (1 mM), and seeded with bacteria, from the RNAi library purchased from Geneservice (Kamath and Ahringer, 2003), verified by restriction enzyme digestion of purified plasmids. A few L3–L4 hermaphrodites of strains transgenic for a *gfp* reporter fusion, were transferred from an area off of the bacterial lawn of an OP50 seeded NGM plate, first to an unseeded NGM plate for a few minutes, and then to the RNAi plates. The RNAi plates were maintained at 20°C for 3 days before observing the progeny. For genes for which RNAi did not cause sterility or developmental arrest, some progeny from the first plate were transferred to a fresh RNAi plate so that phenotypes could also be examined in the subsequent generation. The negative control was HT115 bacteria containing pL4440, as for all clones in the RNAi library, but without an insert between the T7 promoters. A positive control was an equivalent bacterial strain with a pL4440 insert for *unc-22*.

### 2.9 Yeast one- and two-hybrid screens

Yeast one-hybrid (Y1H) and yeast two-hybrid (Y2H) screens and assays were performed as described previously (Deplancke et al., 2004; Reece-Hoyes et al., 2011; Vermeirssen et al., 2007; Walhout, 2006). For Y1H experiments, promoter entry clones for *ceh-14* and *ceh-63* were retrieved from the *C. elegans* Promoterome library and the entry clone for *mbr-1* was generated *ab initio* by Gateway cloning. (See Supplementary Table 1 for primer sequences.) *Promoter::reporter* (*HIS3/lacZ*) bait fusions for *ceh-14*, *ceh-63* and *mbr-1* were generated by Gateway LR recombination reactions between the promoter entry clones and the *pMW2-HIS3* and *pMW3-lacZ* yeast expression vectors. *Promoter::reporter* bait fusions were linearized and transformed into the YM4271(MATa) yeast host strain with integration into the genome by homologous recombination. Twelve clones from each integration were assayed for self-activation of *HIS3* and *lacZ* expression and the clone with the lowest level for each bait was used for subsequent screens. For Y2H experiments, CEH-14 and CEH-63 ORF bait fusions and strains were generated previously in the Walhout laboratory by Gateway cloning of CEH-14 and CEH-63 ORFs into the Gal4DB yeast expression vector pDEST-32 (Invitrogen) and transformation into the MaV103(MATa) host strain.

Screens for transcription factors that interact with a promoter (Y1H) bait or TF (Y2H) bait were performed using the enhanced Y1H (eY1H) approach (Reece-Hoyes et al., 2011). eY1H screens are more efficient than traditional library screens because the transcription factors are presented to the baits as an array of yeast prey strains (Yα1867 for Y1H, Y1Hα001 for Y2H, both MATα), each transformed with a different Gal4AD-TF fusion. 755 *C. elegans* transcription factors are included in the array, with each TF represented four times and thus retested inherently. A bench top robot (RoToR, Singer Instrument, Somerset, UK) was used to precisely transfer the (up to) 1536 yeast colonies present on each media plate. To prepare for screens, bait yeast cells were propagated as lawn cultures in standard RoToR dishes containing YAPD (Y1H) or Sc-Leu (Y2H) media. The transcription factor array was maintained on Sc-Trp media in sets of three 1536-colony plates each containing four copies of up to 384 AD-TF prey yeast clones. To set up a mating, each bait lawn and the transcription factor array were sequentially copied to YAPD plates. The yeast were grown at 30°C for 3 days before being copied to Sc-His-Ura-Trp (Y1H) or Sc-Leu-Trp (Y2H) plates to select for successfully mated diploids. Diploids were grown for two days before being copied to Sc-His-Ura-Trp (Y1H) or Sc-Leu-Trp (Y2H) plus 5 mM (Y1H) or 20 mM (Y2H) 3-AT (3-Aminotriazole) and 80 mg/ml X-gal plates and incubated at 30°C. Plates were monitored over the next 5–7 days for the expression level of the reporters. Colonies that can grow in the absence of histidine, overcome the inhibitory effects of 3-AT, and turn X-gal into a blue compound are expressing the reporters, indicating a transcription factor-promoter (Y1H) or transcription factor–transcription factor (Y2H) interaction. Positives, with at least two of the four colonies containing a particular transcription factor showing reporter expression, were identified according to their array coordinates. The transcription factor ORFs of positive colonies were PCR amplified using primers corresponding to the vector and sequenced to verify the identity of the transcription factors.

To directly test individual transcription factor–promoter (Y1H) and transcription factor–transcription factor (Y2H) interactions, plasmids encoding Gal4AD-TF prey fusions were transformed into haploid promoter::reporter (Y1H) or Gal4DB-TF (Y2H) bait strains and activation of reporters in transformants was assessed with a β-galactosidase assay on overlay filter membranes and from growth on selective plates plus 20, 40, or 60 mM 3-AT (Vermeirssen et al., 2007).

## 3 Results

### 3.1 ceh-63 transcripts

The absence of EST data for *ceh-63* was consistent with the very restricted expression driven by the predicted promoter for this gene, but meant that the predicted exon/intron structure of the gene model was based entirely on the genomic sequence. Nevertheless, we confirmed the gene structure for *ceh-63*, all 5 exons as presented in WormBase, by PCR amplification of cDNA fragments from a *C. elegans* cDNA library preparation (Fig. 1). PCR directed at the homeodomain encoding region of the transcript, however, yielded two differently sized, but equally represented, products. Sequencing revealed the larger product differed in having intron 3 specifically retained. The corresponding cDNA would not encode a complete homeodomain and would have been derived from a probably non-functional, improperly / partially processed transcript. The frequency of intron 3 retention *in vivo* was not directly assessed. PCRs to characterize the ends of the *ceh-63* transcription unit yielded some weak specific bands within a substantial background, consistent with a low abundance of the target transcripts. Sequenced PCR products, cloned after amplification of the start of the transcript, included 0, 206 or 370 nucleotides upstream of the predicted translation initiation codon. Sequence matching the SL1 or SL2 trans-spliced leaders was not found suggesting *ceh-63* is not subject to trans-splicing and is not expressed as a downstream gene in an operon. However, the 370 nucleotide untranslated upstream region included the last 9 nucleotides of the experimentally confirmed 3′UTR of the upstream gene, *C02F12.4*. Therefore, the position where transcription starts for *ceh-63* remains unclear. Direct sequencing of PCR products for the end of the transcript revealed 3′UTRs with either 102 or 106 nucleotides after the termination codon. These two versions were equally represented in the sequencing output suggesting that there are two closely spaced transcription termination points for *ceh-63*. (Independent EST data appeared subsequently, in WormBase WS204, consistent with this transcript analysis and is discussed below.)

### 3.2 *ceh-63* expression pattern

**Fig. 2:**
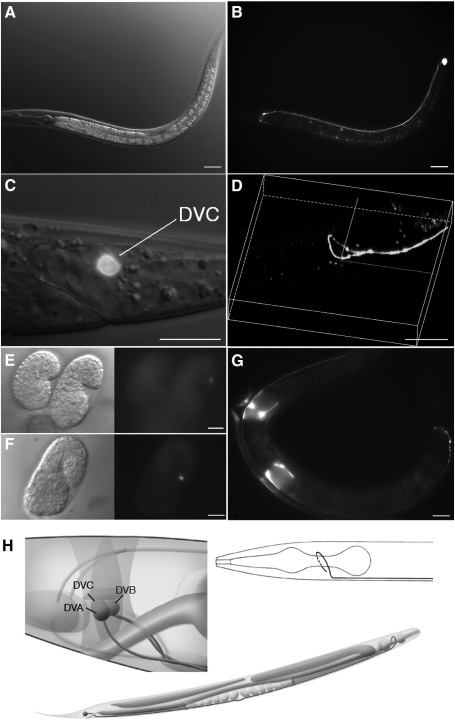
Expression of *ceh-63*^*prom*^*::gfp* in UL2650. GFP was expressed from *ceh-63*^*prom*^*::gfp* (pUL#JRH10H1) in a single nerve cell with its cell body in the tail ganglion and process extending anteriorly to the nerve ring. (A) DIC image of a mid larval stage. (B) Corresponding GFP fluorescence. (C) This cell was confirmed to be the single interneuron DVC by DIC microscopy, the GFP fluorescence superimposed. (D) The axon of DVC forms a ring structure, following round the circumpharyngeal nerve ring, in the head region, as revealed by confocal microscopy. (E, F) Expression was not seen in young embryos and starts in comma stage embryos. (G) GFP also occurs in the uterus in young adult hermaphrodites. (H) The DVC morphology was determined previously from reconstructions of serial electron microscopy sections (White et al., 1986; adapted from WormAtlas). The position in the tail of the cell body of DVC, with respect to those of neurons DVA and DVB, is depicted. The DVC axon in the nerve ring was seen as smooth and non-branching, with a small gap. Bars represent 25 μm in A, B and G, 10 μm in C, E and F, and16 μm in D.

In an earlier project (Reece-Hoyes et al., 2007), the assayed *ceh-63*^*prom*^*::gfp* reporter fusion in pUL#JRH10H1 contained the entire 562 bp upstream intergenic region from the *ceh-63* translation initiation codon to the termination codon of *C02F12.4.* Our transcript analysis confirmed experimentally that this assayed Promoterome fragment (Fig. 1) was appropriate for assessment of *ceh-63* promoter activity. GFP was observed clearly in a single neuron in the tail ganglion (Figs. 2A and B) in hermaphrodites. We identified this cell as the interneuron DVC by differential interference contrast (DIC) microscopy (Fig. 2C). DVC, together with DVA and DVB, are the three unpaired interneurons in the dorsal–rectal ganglion of the *C. elegans* tail, with their cell bodies close together in a readily identified arrangement in the L3 stage. The GFP can be seen in the axon of DVC extending out from the cell body, round the rectum, along the whole body length on the ventral side, and joining the nerve ring in the head (Fig. 2D). Expression of *ceh-63*^*prom*^*::gfp* in DVC can be detected in embryos from the comma stage, shortly after DVC is born at about 340 min after the first cell division (Fig. 2F) and continues throughout the life of the animal. Expression was also observed in uterine wall cells (Fig. 2G) but since the GFP was spread throughout the cells the exact cell identities were difficult to identify with this reporter gene fusion.

In males, in addition to DVC which was seen consistently, up to 4 other cells in the tail expressed *ceh-63*^*prom*^*::gfp* (data not shown). Expression in these additional cells started from the end of the L4 stage, and strengthened into the adult, but was inconsistent. In some of the males, one or 2 expressing cells, in addition to DVC, had GFP labelled axons extending into the ventral nerve cord. In others, the GFP in the additional cells was much weaker and appeared limited to the cell body. Among 26 specifically examined, 3, 6, 9, 6 and 2 males had DVC plus 0, 1, 2, 3 or 4 additional cells, respectively. Because the expression was highly variable and GFP was not shown in earlier stages, specific cell identities were not determined.

**Fig. 3:**
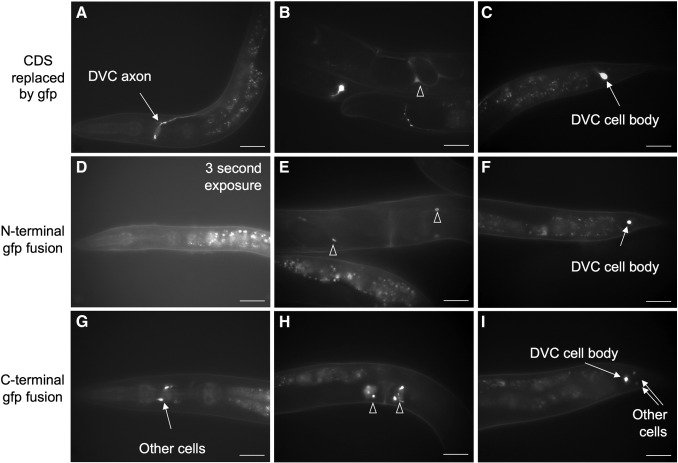
GFP expression of *ceh-63* recombineered reporter gene fusions. (A, B, C) The recombineered fosmid fUL#HF003.1, with the *ceh-63* coding region replaced by *gfp*, drove GFP expression in DVC and in the uterus (open triangle). (D, E, F) The *gfp* reporter inserted immediately after the start codon of *ceh-63* in fosmid fUL#HF001.1, was also expressed in DVC and the uterus (open triangles), but the GFP signal was low and nuclear-localized. Expression of this N-terminal fusion was not observed in the head even with long exposures. (G, H, I) For fUL#HF002.1, with *gfp* inserted immediately before the stop codon of *ceh-63*, low levels of GFP were again observed in DVC and the uterus, at a similar level to that of the N-terminal fusion, but GFP was also observed in a few other cells in both the head and tail. Images presented are for strains: UL3025 (A, B, C); UL3015 (D, E, F); UL3234 (G, H, I). Exposure times were 1.5 seconds, apart from the 3 second exposure of panel D. Bars represent 25 μm.

To confirm the *ceh-63* expression pattern additional *gfp* fusions were constructed by fosmid recombineering. The 562 bp Promoterome fragment may not have contained all the *cis*-acting regulatory elements needed to drive expression of GFP in the same pattern as the endogenous *ceh-63.* However, the fosmid WRM068cD06 contains 14,915 bp upstream and 18,126 bp downstream of *ceh-63* (Fig. 1) and would be expected to include all such regulatory elements. When the reporter was introduced into the fosmid, precisely replacing the entire *ceh-63* coding region (construction fUL#HF003.1), and the recombineered fosmid was used to generate the transgenic strain UL3025, GFP expression was observed in DVC from late embryogenesis through to the adult stage (Figs. 3A and C) and in the uterus (Fig. 3B) in young adult animals, as for the promoter fusion. Expression was still very obvious, but weaker than that driven by just the upstream intergenic region. When *gfp* was inserted into the fosmid precisely after the initiation codon or before the termination codon (fUL#HF001.1 and fUL#HF002.1, respectively) so as to encode N-terminal and C-terminal CEH-63 translational fusions, expression was again observed in DVC and the uterus (Figs. 3D to I). However, the GFP signal was localized to the cell nuclei, presumably due to the DNA binding activity of the transcription factor taking the fusion protein to the nucleus, and expression was at an even lower level than for the two transcriptional fusions. The nuclear localization allowed identification of the uterine cells expressing the CEH-63 C-terminal GFP fusion (Fig. 3H) as ut2 and ut3. This identification was made in young adults after uterine development was complete, when this expression component first appeared, but before embryos distorted the uterus wall. Although no extra expression was observed for the N-terminal fusion, for the C-terminal fusion there was also reliable expression in 6–10 nuclei in the head region and at a similar level to that in DVC (Fig. 3G). This expression pattern component was not observed for any of the other fusions assayed for *ceh-63*. There was also weak but frequent expression of the C-terminal fusion in two other unidentified nuclei, posterior to DVC, in the hermaphrodite tail (Fig. 3I). The significance and origins of the extra expression seen for this particular reporter gene fusion arrangement is unclear and these components were not studied further.

### 3.3 ceh-63 deletion appears to confer a very subtle phenotype

The *ceh-63*(*tm541*) genomic deletion lacks 1209 bp of the 1642 bp transcribed region of *ceh-63*, including almost the whole protein coding region (Fig. 1) and is expected to be a null allele. *ceh-63*(*tm541*) was backcrossed into the N2 wild type background 6 times, using PCR to track the deletion allele, yielding the strain UL3122. Both the original *tm541* containing strain, and UL3122 appeared fully viable and fertile and had no obvious morphological, physiological or locomotory defects. Closer study, in a search for an altered phenotype attributable to the *ceh-63*(*tm541*) deletion, was guided by the gene's expression pattern. Both N2 and UL3122 grow at the same speed, and have similar brood sizes (259±39 vs. 260±21, respectively, n = 12–15 for each) and defecation cycles (42±3.8 vs. 43±2.6 seconds, respectively, n = 5 for each). The slightly longer average life span recorded for UL3122 compared to N2 was not statistically significant (data not shown). Mutant animals responded appropriately to gentle touch with an eyelash on the head or tail. UL3122 males mate successfully and produce cross-progeny with no reduction in male mating efficiency. From these observations, *ceh-63* appears dispensable for *C. elegans* development and biology. It is however possible that, in addition to the deletion allele, a second functional copy of *ceh-63* was present in the genome, remained after back-crossing and was modified so as to be missed in PCR amplification designed to detect undeleted versions of the gene.

To investigate whether, despite the lack of more obvious mutant phenotypes, *ceh-63* is in fact required for DVC specification and development, DVC was visualized in the *ceh-63* deletion background using *ceh-63::gfp* fusions. Extrachromosomal arrays carrying either the plasmid-based *ceh-63*^*prom*^*::gfp* or the fosmid based fusion with *gfp* replacing the *ceh-63* coding region were both crossed into the *ceh-63* (*tm541*) mutant background. No obvious change in GFP expression pattern or strength was observed for any developmental stage (data not shown). In the mutant background, the DVC cell body, as identified from the GFP expression, remained in the correct position in the tail ganglia, as examined by DIC microscopy. The DVC axon still extended into and along the ventral nerve cord and around the nerve ring. This suggests that *ceh-63* has no major essential role in the DVC fate decision or the development of DVC morphology, although the caveat raised above about a possible second functional copy of *ceh-63* in the genome still applies. The uterine expression of *ceh-63::gfp* fusions was not detectably altered in the *ceh-63* mutant background either.

**Fig. 4:**
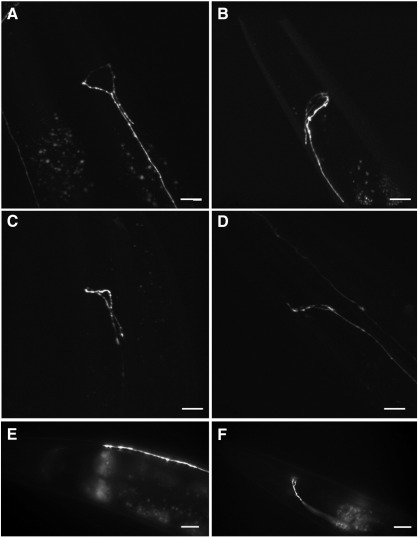
DVC axonal defects in the *ceh-63*(*tm541*) deletion mutant background. The morphology of DVC was observed from *ceh-63*^*prom*^*::gfp* fusion gene expression. (A to D) Various types of defects in the DVC ring could be visualised in projections of confocal microscopy Z-stacks. Other defects, observable with conventional epifluorescence microscopy, included (E) premature termination of the DVC axon and (F) the DVC axon turning back upon itself, forming a small loop without going round the pharynx. Images are for strains UL3551 (A to D) and UL3440 (E and F). UL3551 is described in the text but UL3440 was generated by crossing the transgenic extrachromosomal array *leEx2650*[*ceh-63*^*prom*^*::gfp, unc-119*(*+*)] from strain UL2650 into the background of the independently backcrossed strain UL3156 [*ceh-63* (*tm541*) *X; him-5* (*e1467*) *V*]. Bars represent 16 μm (A to D) or 10 μm (E and F).

Although the DVC cell body and much of the axonal projection were not apparently affected by the *ceh-63* deletion, DVC's projection into the nerve ring was often abnormal (Table 1). In adult hermaphrodites, in the wild type background, the DVC axon almost completely encircles the pharynx in the nerve ring, smoothly, as revealed by confocal microscopy (Fig. 2). In the *ceh-63* mutant background various types of DVC nerve ring defect were often observed including: the DVC axon extending only halfway round the nerve ring; the axon encircling the pharynx incompletely, with gaps of various extents; the axon branching in the nerve ring (Fig. 4B); the axon extending too far, beyond the position where the axon entered the nerve ring (Fig. 4A) and crossing over itself (Fig. 4C); the axon terminating prematurely before reaching the nerve ring (Fig. 4E); the axon seeming to meander in the nerve ring (Fig. 4F); and, occasionally, the axon in the nerve ring appearing totally disorganized (Fig. 4D). As some of these defects were also observed, although much less frequently, in a wild type background and rescue of the phenotype has not been demonstrated with the cloned *ceh-63* gene, the DVC axon guidance problems are not confirmed as a *ceh-63* mutant phenotype.

### 3.4 No function could be ascribed to the nerve cell DVC itself

While inactivation of *ceh-63* did not appear to cause a major perturbation of DVC morphology and there were no obvious consequences for *C. elegans* behaviour, DVC function may still have been severely compromised in the deletion mutant. In two previous studies (Durbin, 1987; Li et al., 2006), DVC was ablated with a laser microbeam and no effects on *C. elegans* survival or development were observed. We repeated these DVC ablations but in UL2650 individuals bearing *ceh-63*^*prom*^*::gfp*. DVC was successfully ablated in eight L1s as judged by the absence of a GFP labelled DVC cell body after three days of growth, although one to three short sections of GFP labelled axon remained along the body of all eight. The animals lacking DVC, when examined as larvae or adults, had no apparent difference from mock-ablated or unmanipulated individuals. No abnormalities in morphology or locomotion were observed. Therefore, the DVC neuron appears dispensable for *C. elegans* in normal laboratory conditions and even if the *ceh-63* deletion had completely destroyed DVC function this may not have been apparent in our characterization of the mutant.

Whilst removal of DVC activity may have had no effect because of compensation by the rest of the nervous system, inappropriate activation might have elicited a behavioural response to reveal the function of DVC. Expression of channelrhodopsin-2 (ChR2), a light-gated cation channel from the green alga *Chlamydomonas reinhardtii*, in DVC would allow controlled activation of this nerve cell. ChR2 has been used to activate excitable cells in *C. elegans* and thus to evoke rapid behavioural responses, simply in response to blue light stimulation (Boyden et al., 2005; Nagel et al., 2003, 2005). The DVC-specific *ceh-63* promoter made it feasible to specifically target ChR2 to DVC without interfering with other nerve cells. Despite controls demonstrating that the experimental system was working as intended (See Material and methods Section 2.7) no specific behavioural response was observed in adult hermaphrodites, upon blue light activation of ChR2::YFP expressed in DVC. No movement was initiated immediately upon applying the light. As DVC synapses with the locomotory command interneurons AVA and AVB, forward and reverse locomotion was examined closely. Under constant illumination, animals did start to move forwards or backwards when light was directed at the tail or head respectively, but the same reaction was observed in control animals. *C. elegans* does possess a light sense modality (Edwards et al., 2008; Ward et al., 2008) and the accelerated locomotion in response to blue light observed here was attributed to this phototaxis rather than a specific reaction to stimulation of DVC. Specific stimulation of DVC failed to reveal the function of this interneuron.

### 3.5 Genetic regulation between *ceh-14*, *ceh-63* and *mbr-1* in DVC

DVC morphology suggests this is a fully functional nerve cell with its development dependent upon appropriate gene expression in which *ceh-63* might be involved. We sought to identify regulatory interactions involving *ceh-63*. First we examined the reporter expression in transgenic strains containing reporter gene fusions for all the transcription factor genes (*lin-11*, *ceh-14* and *mbr-1*) specifically reported as being expressed in DVC and a few other locations (Cassata et al., 2000; Kage et al., 2005; Sarafi-Reinach et al., 2001). LIN-11 and CEH-14 have LIM-type homeodomains while MBR-1 contains two helix–turn–helix motifs. In the initial strains, with a wild type copy of *ceh-63*, reporter gene fusion expression in DVC was confirmed for two of the three transcription factor genes, *ceh-14* and *mbr-1*. After crossing, to place the reporter gene fusion for *ceh-14* into the *ceh-63* mutant background, DVC expression was apparently unaffected. This suggests that *ceh-14* DVC expression is independent of *ceh-63*, although the caveat mentioned above about a second functional copy of *ceh-63* remains.

**Fig. 5:**
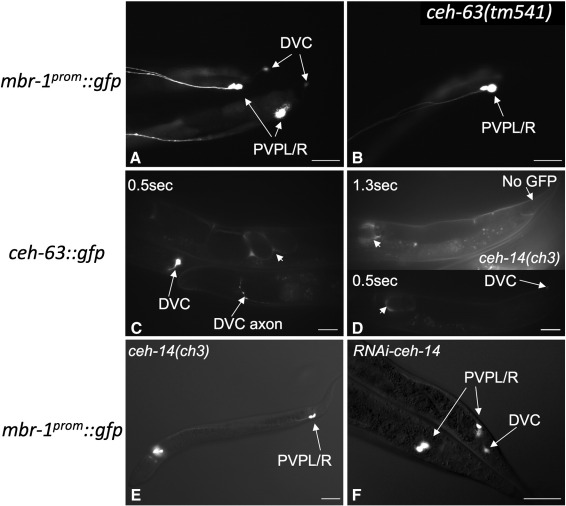
CEH-63 and CEH-14 activate *mbr-1* expression and CEH-14 activates *ceh-63* expression, in DVC. (A) *mbr-1*^*prom*^*::gfp* drives GFP expression in PVPL/R and DVC in the tail region. (B) GFP expression driven in DVC by *mbr-1*^*prom*^*::gfp* was completely abolished in the *ceh-63* deletion mutant background. (C) The recombineered fosmid fUL#HF003.1, with the *ceh-63* coding region replaced by *gfp*, drove GFP expression in DVC and in the uterus (small arrows) (D) GFP expression from fUL#HF003.1 in DVC, but not that in the uterus (small arrows), was either abolished (upper panel)) or greatly reduced (lower panel) in the *ceh-14* (*ch3*) mutant background. (E) *mbr-1*^*prom*^*::gfp* expression in DVC was completely abolished in the *ceh-14*(*ch3*) mutant background. (F) Knock-down of *ceh-14* by RNAi could also eliminate the expression of *mbr-1*^*prom*^*::gfp* in DVC but inconsistently; the lower worm lacks while the upper worm retains DVC expression. Strains generated for this analysis, and imaged here, are UL3074 (B), UL3025 (C), UL3214 (D) and UL3213 (E). The original strain bearing the *mbr-1*^*prom*^*::gfp* transgene (A and F) was described previously (Kage et al., 2005). Bars represent 25 μm.

In contrast, CEH-63 does appear to be required for *mbr-1* expression in DVC. When the chromosomally integrated *mbr-1*^*prom*^*::gfp* transgene was crossed into the *ceh-63*(*tm541*) mutant background, GFP expression in DVC was lost entirely while expression in PVPL/R and head neurons was unaffected (Fig. 5B). DVC itself remained as judged by DIC microscopy. These data imply that *ceh-63* has indeed been disrupted by the *tm541* deletion and suggest that CEH-63 is indispensible for the expression of *mbr-1* in DVC. Formal proof that the *ceh-63*(*tm541*) deletion is responsible for this phenotype, however, would depend on reversion upon transformation with the cloned gene. The converse does not apply; MBR-1 is not required for expression of *ceh-63*. When the *ceh-63*^*prom*^*::gfp* transgene was crossed into the *mbr-1*(*qa5901*) deletion mutant background, the expression of this reporter fusion in DVC was not affected. CEH-63 appears to act, either indirectly or directly, upstream of *mbr-1* in DVC.

Although *ceh-14* expression is apparently independent of CEH-63, CEH-14 is partially required for *ceh-63* expression in DVC. When the transgene bearing the fosmid with *gfp* replacing the *ceh-63* coding region was crossed into the *ceh-14*(*ch3*) mutant background, GFP expression in DVC was abolished or greatly attenuated (Fig. 5D). Amongst 50 adult animals specifically counted, DVC expression was absent from half. The CEH-14 regulation of *ceh-63::gfp* was specific to DVC as the uterus expression component was unaffected. Since the *ceh-63::gfp* DVC expression was not eliminated from every individual, CEH-14 does not appear absolutely essential for *ceh-63* expression, implying that other factors or pathways act redundantly with CEH-14 to activate expression of *ceh-63* in the wild type, albeit weakly.

CEH-14 is absolutely required for *mbr-1* expression in DVC. As CEH-14 partially regulates *ceh-63*, which in turn appears indispensible for *mbr-1* expression in DVC, it was anticipated that inactivation of CEH-14 should at least partially reduce the expression of *mbr-1* in DVC. However, when the chromosomally integrated *mbr-1*^*prom*^*::gfp* transgene was crossed into the *ceh-14*(*ch3*) mutant background, *gfp* expression in DVC was completely abolished, a more severe result than expected (Fig. 5E). This suggests that either even a reduction in *ceh-63* expression is sufficient to eliminate DVC expression of *mbr-1* or there is another route by which CEH-14 controls *mbr-1* expression, as well as *via* activation of *ceh-63*. The latter interpretation is favoured as inactivation of *ceh-14* by RNAi resulted in loss of *mbr-1*^*prom*^*::gfp* DVC expression from 73% (n = 150) of the population (Fig. 5F), with no apparent effect on *ceh-63*^*prom*^*::gfp* expression.

CEH-14 does not seem to regulate its own expression in DVC. When the *ceh-14::gfp* fusion was crossed into the *ceh-14*(*ch3*) mutant background *gfp* expression in DVC remained unchanged. And, as reported above, CEH-63 does not seem to regulate its own expression in DVC either, as *ceh-63::gfp* expression in DVC is unaffected in the *ceh-63*(*tm514*) mutant background.

**Fig. 6:**
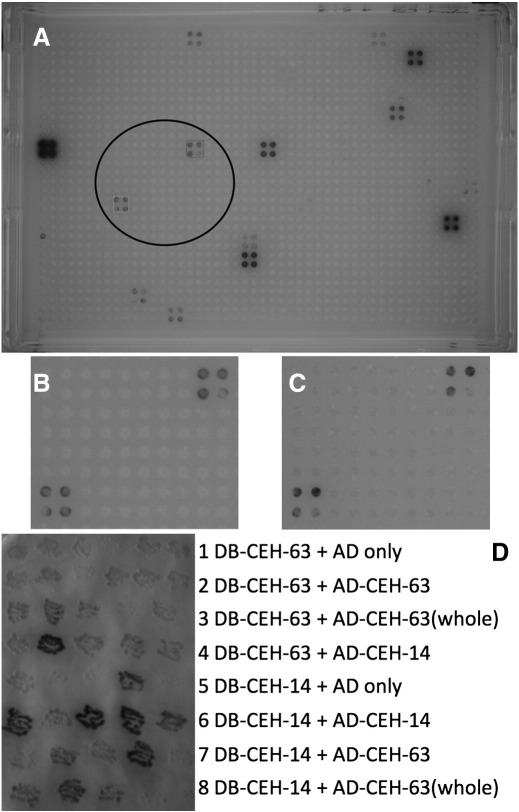
CEH-63 and CEH-14 bound the *mbr-1* promoter in Y1H screens and each other in Y2H assays. (A) One entire Y1H screen plate for the *mbr-1* promoter bait yeast strain mated with the AD-TF array. Each AD-TF fusion is represented by 4 yeast colonies, each representing an independent assay. The assays for AD-CEH-63 and AD-CEH-14 are contained within the circle and show *lacZ* signals (blue) that are very clear, although weaker than for some other AD-TF fusions. (B) The circled region is enlarged for clarity. (C) The results are reproducible as demonstrated by this enlargement of a corresponding region from a repeat of the same Y1H screen. (D) One set of Y2H assays for CEH-63 and CEH-14. A set of six independent patch assays are presented for each of eight Y2H combinations (1–8). The DB-CEH-63 or DB-CEH-14 fusion was tested against the AD alone or the AD-CEH-14 from the TF array or the AD-CEH-63 from the TF array or the specifically constructed AD-CEH-63(whole). The AD-CEH-63 from the TF array lacks the N-terminus of the native CEH-63 while the AD-CEH-63(whole) includes the whole CEH-63 protein. Positive interactions are indicated by the blue colouration.

CEH-14 and CEH-63 both bind directly and independently to the *mbr-1* promoter. When the *mbr-1* promoter was tested against the transcription factor array (Reece-Hoyes et al., submitted for publication; Vermeirssen et al., 2007) in a Y1H screen, two of the 30 positive transcription factors corresponded to CEH-14 and CEH-63 (Fig. 6). The *lacZ* signals for the CEH-14 and CEH-63 interactions with the *mbr-1* promoter were clear although weaker than for CEH-9, COG-1a, DAC-1, DIE-1, LIN-39, MLS-2, ODR-7 and VAB-3a. An interaction with CEH-14 and CEH-63 was subsequently observed in a replicate assay using an independent yeast strain carrying the *mbr-1*^*prom*^*::reporter* fusion, demonstrating the results were reproducible. Direct transformation of the *mbr-1*^*prom*^*::reporter* bait strain with the AD-CEH-14 and AD-CEH-63 prey plasmids further verified these interactions. Along with the genetic regulatory relationships presented above, CEH-14 and CEH-63 are proposed to be direct positive regulators of *mbr-1* in DVC.

Two replicate Y1H screens and direct Y1H assays gave no evidence for CEH-14, CEH-63 or MBR-1 binding to either of the *ceh-14* or *ceh-63* promoters.

CEH-14 did appear to interact with itself and with CEH-63 in direct Y2H assays (Fig. 6D). A DB-CEH-14 bait gave a clearly positive signal for interaction with the AD-CEH-14 prey in 27 of 39 independent assays carried out for individual yeast transformant colonies. The DB-CEH-14 bait also interacted with the AD-CEH-63 prey from the transcription factor array but only in 13 of 42 such assays. Confidence in these interactions is reduced by the weak signal observed occasionally for the DB-CEH-14 bait when assayed against a non-fusion AD domain only control prey (5 in 20 assays). However, the AD-CEH-63 prey from the transcription factor array is missing the N-terminus of the CEH-63 protein. When a new Gal4AD prey fusion was specifically constructed with a full length CEH-63 and assayed against the DB-CEH-14 bait, 38 of 45 assays were positive and the strength of signal was consistently stronger than with the incomplete version of the DB-CEH-63 prey. This suggests that CEH-63 does interact strongly with CEH-14, but a strong interaction depends on the N-terminus being intact.

## 4 Discussion

Transcript analyses have verified the annotated exon/intron structure of *ceh-63*, confirming that this gene is transcribed and revealing that the reporter gene fusion assayed previously (Reece-Hoyes et al., 2007) would have been appropriate to reveal *ceh-63* promoter activity. While the 3′ end of the gene appears well defined, the 5′ end is less so. *ceh-63* cDNAs were identified with 0, 206 and 370 bp of 5′ UTR. *ceh-63* ESTs now in WormBase have 230 bp, 144 bp and 113 bp of 5′ UTR. These could represent various versions of mature transcripts resulting from alternative transcription start points / promoters, or be simply the result of incomplete first strand cDNA synthesis. Given the simplicity of the expression pattern with apparently just two distinct expression pattern components, more than two alternative promoters would seem unlikely. In addition, the cDNA with the longest 5′ UTR overlaps the transcribed region of the gene immediately upstream and it is unclear how such a transcript could arise. Even for the other cDNAs, there is little room for the *ceh-63* promoter in the upstream intergenic region.

The main components of the *ceh-63* expression pattern, as revealed by multiple reporter gene fusion arrangements, are DVC and uterus. However, additional expression in cells in the head and tail, particularly the male tail, was observed weakly and inconsistently, but repeatedly. The impression is given of repression of expression of *ceh-63* in the additional cells being unreliable, as if such expression can be tolerated and does not need to be fully suppressed. Perhaps some of the transcriptional control system(s) activating *ceh-63* in DVC is shared with these other nerve cells. Nevertheless DVC and the uterus are likely to be the primary sites of *ceh-63* function.

Despite *ceh-63* appearing to be a fully functional gene, encoding a conserved homeodomain transcription factor, no obvious abnormalities were found in strains with almost the entire protein-coding region deleted. No consequences were observed for locomotion, response to touch, egg-laying, brood size, defecation, growth rate, lifespan or male mating efficiency. *ceh-63* is expressed in the uterus and there could still be minor defects in uterus morphology or development in *ceh-63* mutants. The male tail is involved in complex behaviours for mating and, given the additional *ceh-63* expression in nerve cells in the tail particularly in the male, subtle effects on male mating behaviour may exist. But the strongest *ceh-63* expression is in the interneuron DVC. DVC has most synaptic or gap junction connections (Chen et al., 2006; White et al., 1986) with the backward locomotion command interneuron AVA, the motor neurons AVL and DVB required for anterior body contraction and expulsion muscle contraction in the defecation motor program, and the VC egg-laying motor neurons. Yet all of these behaviours appeared normal in *ceh-63* mutants. However, no behavioural consequences were observed either for laser ablation or optogenetic activation of DVC. Therefore, there might be redundancy for DVC activity amongst nerve cells of the nervous system or DVC function is not important under the conditions of the assays. DVC pre-synaptic partners are mainly interneurons and so DVC could have a role in higher level functioning of the nervous system. Even if *ceh-63* deletion completely destroyed DVC function, consequences would probably not have been detected.

**Fig. 7:**
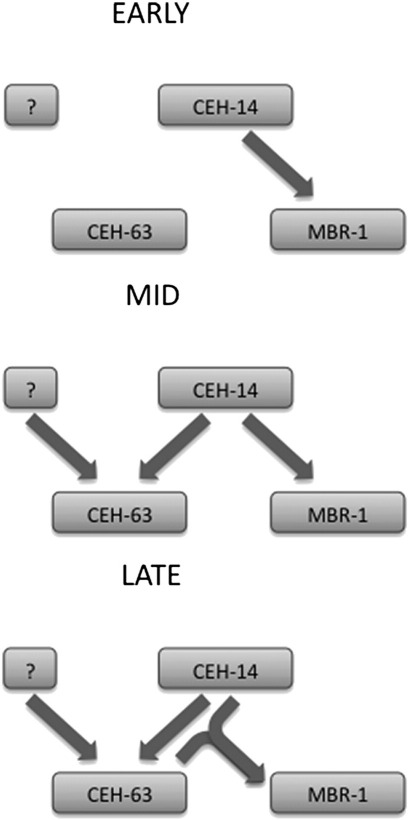
A model for the potential CEH-14/CEH-63/MBR-1 transcription factor network in DVC. Regulatory interactions occur successively, as represented from early to mid to late time points, as the embryo is elongating. First CEH-14 activates *mbr-1*. Subsequently *ceh-63* is activated by CEH-14 (not necessarily directly) and another unknown pathway (denoted by ?), either being partially sufficient. Finally CEH-14 and CEH-63 activate *mbr-1* co-operatively. In the *ceh-63* mutant, CEH-63 is not produced and so the activation of *mbr-1* is not sustained. In the *ceh-14* mutant, there is no initial activation or sustained activation of *mbr-1*, but CEH-63 can be activated weakly/unreliably by the alternative pathway. Upon *ceh-14* RNAi, the early expression of CEH-14 and the initial activation of *mbr-1* fail. Although the later expression of CEH-14 remains and is sufficient to activate CEH-63, this is not sufficient to initiate *mbr-1* expression.

Although *ceh-63* may be dispensable under laboratory conditions, a transcription factor regulatory network operating in DVC through CEH-63 was defined, from expression pattern analyses and yeast one-hybrid assays. CEH-14 is involved in *ceh-63* activation, while CEH-14 and CEH-63 are suggested to directly activate *mbr-1* expression, in DVC (Fig. 7). This network resembles a coherent feed-forward loop, a very common motif in transcriptional regulatory networks (Gerstein et al., 2010; Zhu et al., 2007).

Complete loss of *ceh-63* expression from DVC in only half the individuals of *ceh-14* mutant strains suggests CEH-14 is involved in *ceh-63* activation but alternative, unreliable routes to weak *ceh-63* activation are also operational. The lack of CEH-14 binding to the *ceh-63* promoter in yeast one-hybrid assays and the absence of CEH-14 binding sites in the *ceh-63* promoter in ChIP-seq analysis (Gerstein et al., 2010), suggest CEH-14 may not activate *ceh-63* directly. There is no information on the identity of any intermediate transcription factor but such a factor could also mediate the CEH-14 independent activation of *ceh-63*.

Absence of either CEH-14 or CEH-63, in deletion mutants, appeared to cause the complete loss of *mbr-1* expression in DVC. The simplest model would be for CEH-14 and CEH-63 to interact as a heterodimer to bind to and activate the *mbr-1* promoter. Although CEH-14 and CEH-63 were shown, in yeast one-hybrid assays, to bind the *mbr-1* promoter individually, presumably as monomers or homodimers, a CEH-14/CEH-63 interaction was also demonstrated in yeast two-hybrid assays. The concentrations of these two proteins *in vivo* and the affinities of the homo- and heterodimers for the *mbr-1* promoter may be crucial factors. A lower affinity of the CEH-14 homodimer for the *mbr-1* promoter may avoid *mbr-1* being expressed wherever *ceh-14* is expressed while a higher affinity of the CEH-14/CEH-63 heterodimer could allow transcriptional activation specifically in DVC. Multiple sites of CEH-14 binding were mapped to the *mbr-1* promoter by the modENCODE consortium (Gerstein et al., 2010).

Remarkably, *ceh-63*(*RNAi*) failed to affect *mbr-1* expression, while *ceh-14*(*RNAi*) did affect *mbr-1* expression but without affecting *ceh-63* expression. As the *C. elegans* nervous system is normally resistant to RNAi, the role of CEH-14 in *mbr-1* expression that is revealed by RNAi may operate before DVC's refractility to RNAi arises during embryogenesis, with CEH-14 activation of *ceh-63* not occurring until afterwards. The role of CEH-63 in *mbr-1* activation would then also occur afterwards, consistent with the lack of effect of *ceh-63*(*RNAi*) on *mbr-1* expression. Perhaps the earlier activation of *mbr-1* by CEH-14 is a pre-requisite for subsequent maintenance of *mbr-1* expression by CEH-63, with the later activation of *ceh-63* expression also dependent on CEH-14.

CEH-14 appears to have a key upstream role in the development of several nerve cells beyond DVC, including AFD and PVT. The exclusive expression of *gcy-8* and *gcy-18* in AFD is down regulated by inactivating either *ceh-14* or *ttx-1*, and completely abolished in the *ceh-14;ttx-1* double mutant (Kagoshima et al., 2009). Similarly, CEH-14 and LIM-6 together define *zig* expression in PVT (Aurelio et al., 2003). However, in *ceh-14* mutants, AFD and PVT still adopted the correct fates and simply failed to express certain differentiation markers, with electron microscopy being needed to reveal minor dendritic defects in AFD's fingers (Aurelio et al., 2003; Cassata et al., 2000). The only defect found previously in *mbr-1* mutants was an effect on the number of neurites on AIM interneurons in the nerve ring (Kage et al., 2005) and this may have relevance for the possible minor axonal defects reported here for *ceh-63* mutants. No downstream terminal differentiation genes have been identified for the DVC transcription factor network defined here. In the *ceh-14*(*ch3*) mutant, like *ceh-63* and *mbr-1* mutants, consequences for DVC may only be subtle; although affects on DVC axon morphology have not been assessed, the DVC nucleus appeared normally positioned. Such observations may be typical, reflecting the complications of transcription factor regulatory networks as they operate in nerve cells and direct the complex cellular morphology of this cell type.

Within the *C. elegans* genome, *ceh-63* is most homologous (E value < e^-10^) to *ceh-7* (C34C6.8, 49% identity, 67% similarity) and *R06F6.6* (47% identity, 70% similarity). *ceh-7* and *R06F6.6* appear to be expressed in a limited number of nerve cells (Kagoshima et al., 1999; Reece-Hoyes et al., 2007), like *ceh-63*, but not in DVC. One of the cells expressing the *R06F6.6*^*prom*^::*gfp* reporter fusion (Reece-Hoyes et al., 2007) was DVA (data not shown), which is anatomically adjacent to and with similar morphology to DVC. This similarity may reflect a conserved homologous functional connection between the *ceh-63* and *R06F6.6*. Nevertheless, reciprocal BLASTP searches suggested that both *ceh-63* and *R06F6.6* have distinct orthologues (E values between e^− 50^ to e^− 90^, 60–90% similarity) in both *Caenorhabditis remanei* and *Caenorhabditis briggsae*. There is a *ceh-7* orthologue in *C. briggsae*, but the *ceh-7* orthologue seems to be missing from *C. remanei.* These orthologous genes appear to have been maintained in parallel at least since the divergence of these species in the evolutionary history of this genus.

It seems remarkable that the biological function of a transcription factor such as CEH-63, conserved over many millions of years of evolution with direct orthologues in other *Caenorhabditis* species, and the role of DVC, in which this protein seems to be primarily expressed in *C. elegans*, should be so difficult to discern. This might be a reflection of the robust properties of neural and transcription factor networks across the animal kingdom. CEH-63 appears to be a typical homeobox protein and DVC appears to be a typical nerve cell. Robustness, possibly resulting from network based redundancy, appears to be a general characteristic of transcription factor networks, as many transcription factor genes fail to yield obvious phenotypes when inactivated, and may well apply to neural networks. If so, it will be important to find novel approaches with which to reveal the biological roles of such transcription factors and nerve cells.

The following are the supplementary materials related to this article.

Supplementary Table 1 PCR primers.

## Figures and Tables

**Table 1:** Comparison of DVC axon defects in UL2650 (*ceh-63* wild type) and UL3551 (*ceh-63* mutant) adult hermaphrodites.

DVC axonal extension defect type	UL2650 (*ceh-63*^*+*^)	UL3551 (*ceh-63*^*−*^)
Extension only halfway round the nerve ring	3	21
Incomplete, with a small gap in the nerve ring	18	8
Branching in the nerve ring	1	6
Premature termination, not reaching the nerve ring	2	1
Too long, crossing over itself	3	26
Meandering in the nerve ring	0	23
Totally disorganized	0	2
Normal with no defect apparent	132	31

Total defects	27	87
Total examined	159	118
Percentage with defects	17%	73.7%
