# Supplementary material for: A regulatory cascade of three transcription factors in a single specific neuron, DVC, in Caenorhabditis elegans
Source: Gene. 2012 Feb 15;494(1):73–84. doi: 10.1016/j.gene.2011.11.042 (PMC3271191; doi:10.1016/j.gene.2011.11.042)
Supplement: Supplementary Table 1 — PCR primers. [file mmc1.doc]

**Supplementary Table 1.** PCR primers

| **Usage** | **Name** | **Sequences** |
| --- | --- | --- |
| recombineering gfp fusions | CEH-63-GFP-  For (after ATG) | caattgctctgagctttcattgcccctgttttgctcattgtgttgttagcATGAGTAAAGGAGAAGAACT |
|  | CEH-63-GFP-  Rev (after ATG) | aagggaaggttaaaaaaataatattatttcaaacttaccgttttagacgtTTTGTATAGTTCATCCATGC |
|  | CEH-63-GFP-  For (before STOP) | ggccctcgcattttgcatattcacttccttctgatcaacagaacaatgttATGAGTAAAGGAGAAGAACT |
|  | CEH-63-GFP-  Rev (before STOP) | tttcatgaagaattcagtataaaaaagtagacaaagagctgcaaaaatcaTTTGTATAGTTCATCCATGC |
| *mbr-1* Gateway cloning | attB4-mbr-1promL-For | GGGGACAACTTTGTATAGAAAAGTTGTAggcgaaagagtaagattaagtgc |
|  | attB1r-mbr-1prom-Rev | GGGGACTGCTTTTTTGTACAAACTTGTcattgtacctacgaaaatacaa |
| transcript analysis | pPC86 vector-For | TTCAAAACCACTGTCACCTG |
|  | pPC86 vector-Rev | CCAACGATTTGACCCTTTTC |
|  | CEH-63-cDNA-For1 | AGGAGCAATCGACCGACTAA |
|  | CEH-63-cDNA-Rev4 | GATTTTGTGGCGAAGGAGAA |
|  | CEH-63-cDNA-Rev3 | GTTCTCCGGTTCTGAAACCA |
|  | CEH-63-exon3-Rev2 | AAGTTGTTCTTTTAGTCGGTCGAT |

For recombineering primers, for fusions at the start (after ATG) or end (before STOP) of the *ceh-63* protein-coding region, the gene-specific sections are in lower case and those corresponding to the ends of the *gfp* reporter are in upper case, for priming in the forward (For) or reverse (Rev) direction with respect to transcription. For the Gateway cloning primers, the gene specific sections are in lower case and the *att* recombination site sections are in upper case.
